# Polyamide 11 Composites Reinforced with Diatomite Biofiller—Mechanical, Rheological and Crystallization Properties

**DOI:** 10.3390/polym15061563

**Published:** 2023-03-21

**Authors:** Marta Dobrosielska, Renata Dobrucka, Dariusz Brząkalski, Paulina Kozera, Agnieszka Martyła, Ewa Gabriel, Krzysztof J. Kurzydłowski, Robert E. Przekop

**Affiliations:** 1 Faculty of Materials Science and Engineering, Warsaw University of Technology, ul. Wołoska 141, 02-507 Warsaw, Poland; 2 Department of Non-Food Products Quality and Packaging Development, Institute of Quality Science, Poznań University of Economics and Business, al. Niepodległości 10, 61-875 Poznań, Poland; 3 Centre for Advanced Technologies, Adam Mickiewicz University in Poznań, ul. Uniwersytetu Poznańskiego 10, 61-614 Poznań, Poland; 4 Faculty of Mechanical Engineering, Bialystok University of Technology, ul. Wiejska 45c, 15-351 Bialystok, Poland

**Keywords:** polyamide 11, diatomaceous earth, diatoms, silanization, silanes, surface properties, mechanical strength, hydrophilic composite system

## Abstract

Amorphic diatomaceous earth is derived from natural sources, and polyamide 11 (PA11) is produced from materials of natural origin. Both of these materials show a low harmfulness to the environment and a reduced carbon footprint. This is why the combination of these two constituents is beneficial not only to improve the physicochemical and mechanical properties of polyamide 11 but also to produce a biocomposite. For the purpose of this paper, the test biocomposite was produced by combining polyamide 11, as well as basic and pre-fractionated diatomaceous earth, which had been subjected to silanization. The produced composites were used to carry out rheological (melt flow rate-MFR), mechanical (tensile strength, bending strength, impact strength), crystallographic (X-ray Diffraction-XRD), thermal and thermo-mechanical (differential scanning calorimetry–DSC, dynamic mechanical thermal analysis–DMTA) analyses, as well as a study of hydrophobic–hydrophilic properties of the material surface (wetting angle) and imaging of the surface of the composites and the fractured specimens. The tests showed that the additive 3-aminopropyltriethoxysilane (APTES) acted as an agent that improved the elasticity of composites and the melt flow rate. In addition, the produced composites showed a hydrophilic surface profile compared to pure polylactide and polyamide 11.

## 1. Introduction

Filled polymer composites have been successfully applied for many years in various sectors of the economy, such as automotive, aerospace and construction industries [1,2,3]. The most popular fillers include wood flour [4,5], carbon fibres [6,7], glass fibre [8,9], clays [10,11], white titanium [12,13], cellulose [14,15], basalt [16,17] and flax fibre [18]. However, the most important vice of conventional polymer products is their harmful impact on the natural environment, which is a result of ineffective waste management and the growing consumer demand for new products. Even so, the marketing of new, advanced materials based on biopolymers faces many obstacles and has been broadly discussed in the literature by researchers with both academic and industrial backgrounds. It has consistently been shown that an additive to mineral and fibrous fillers has a positive impact on mechanical properties, thermal stability and abrasion resistance, and in many cases, it helps considerably reduce production costs, which is important for many entrepreneurs. Such a cost-effective mineral filler is diatomaceous earth (EUR 0.80/kg on average). In addition to the favourable impact on the mechanical or rheological properties of polymer composites, diatomaceous earth is also used as a filtration medium to remove harmful substances, and its porous structure makes it easy to modify its surface [19].

The use of inorganic fillers in polymer composites entails not only benefits but also challenges. This is because the final properties of the designed materials depend on the interaction (adhesion) at the interface between the filler and the polymer matrix. The improved interactions at the filler–polymer interface are an important issue for researchers.

Silanization is a well-known method for improving adhesion between different materials, which is also well described in the literature. Silanes are hybrid compounds that consist of the organic and inorganic parts with a general formula (X(CH_2_)_n_SiR_3_, with ‘n’ usually being 0–3, which makes them applicable as the promoters of adhesion between materials dissimilar in terms of the chemical structure, such as ceramics–polymer and polymer–metal [20,21,22]. The hybrid, inorganic and organic structures of silanes allow us to form stable chemical bonds between organic and inorganic materials. The organic part of silane consists of groups that are chemically reactive or compatible with organic materials (depending on the choice of X substituent and the materials to be compatibilized), such as polymers (e.g., amine, epoxy or vinyl groups). The R substituents are groups susceptible to hydrolysis (alkoxy or chloride groups) responsible for forming siloxane links with the surface of inorganic materials [23].

**Figure 1:**
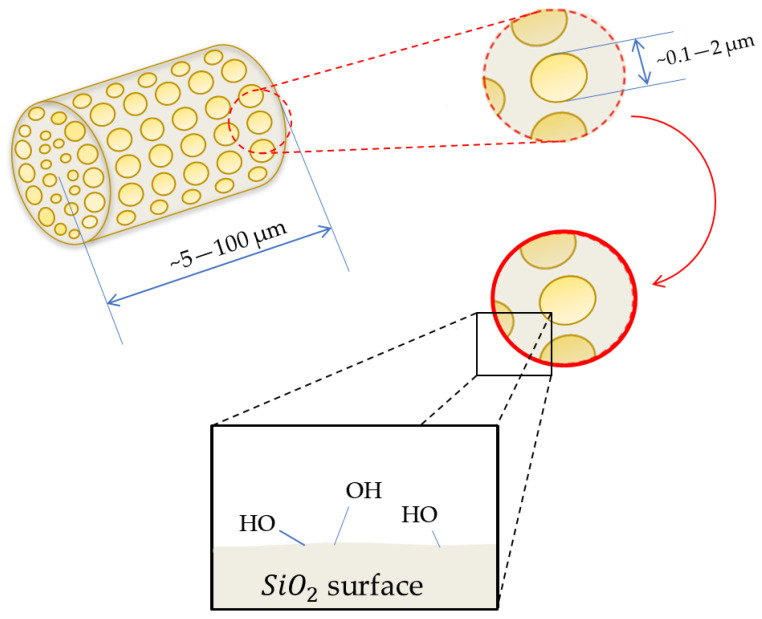
Diagram representing diatomaceous earth microstructure and surface chemistry.

**Figure 2:**
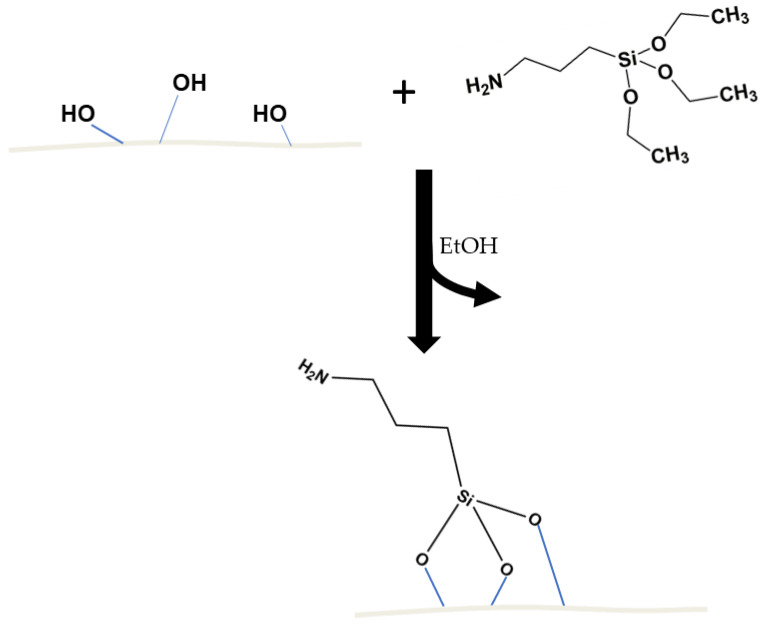
Diagram representing the diatomaceous earth surface silanization process with APTES.

Silane-based couplers form a thin film between incompatible materials, thus improving the interface interactions in composites, which in turn translates into improved mechanical properties of the filled polymeric composites. When placed on an inorganic surface, silanol oligomers react with one another to create branched hydrophobic siloxane bonds -Si-O-Si-. They form metal–siloxane bonds -Si-O-M- (where M = metal) on the surface of the inorganic matrix (for example, silica, diatomaceous earth or metal oxides containing hydroxyl groups, –OH). Figure 1 shows a diagram representing the structure of a single diatom particle comprising diatomite earth, while Figure 2 shows a diagram of its surface silanization with APTES. More details on the microstructure of diatomite have been discussed in our previous works [24], as well as the silanization thereof [25].

Composites based on the PA11 matrix, loaded with silanized fillers for reinforcement, are not commonly known. The literature has recorded few works devoted to PA11 with silanized fillers. We know the methods of strengthening polyamide 11 with organically modified clay [26], organically modified layered silicate (Cloisite 30B) [10], silane (3-aminopropyltriethoxysilane (APTES)) grafted titanate nanotubes [27], and Palygorskite nanofiber clay silanized by the same silane (APTES) [28]. Adding silanes is aimed mainly at improving the interface interactions between the filler and polyamide 11. Additionally, a natural filler is added to produce a biocomposite whose ingredients are derived from renewable sources that can be strengthened and, consequently, we can improve its tensile strength, impact strength or Young modulus. A key point in terms of the industrial application of the PA11/filler composite is its weather resistance, which has been obtained in, for example, the work of Cong et al., which used jute fibres silanized by vinyltrimethoxysilane (VTMOS) [29].

As we had no references in the bibliographical references regarding the use of silanized diatomaceous earth as a filler for polyamide 11, we attempted to produce such composites. In our previous work, we also performed the silanization of diatomaceous earth and examined the impact of the modification on the physicochemical and mechanical properties of composites on the polylactide matrix [25]. Tests have shown that, depending on the silane applied, it is possible to modify individual parameters; for instance, methyltrimethoxysilane causes the greatest growth in processing properties (MFR), n-octyltriethoxysilane reduces the composite degradation, and (3-glycidyloxypropyl)trimethoxysilane helps inhibit the penetration of UV rays into the composite, so the degradation progresses only down to a specified depth. The use of 3-aminopropyltriethoxysilane as a diatomaceous earth modifier may further improve the physicochemical parameters of composites, but this time, for a broader analysis, the composite matrix was polyamide 11 acquired from renewable sources.

In this work, we carried out a chemical modification (silanization) of basic diatomaceous earth pre-fractionated with the use of sedimentation. The prepared material was used as a filler for polyamide 11 to produce composites with a diatomaceous earth content of 2.5, 5, 10 and 20% by weight with a 1% addition of APTES. The produced composites were tested for the impact of silanization on their mechanical and processing characteristics. Furthermore, we compared the thermal and thermomechanical properties of the composites (DSC and DMTA) and tested their surfaces and fractures with an optical microscope. The degree of dispersion of a filler in a polymer was determined using a scanning electron microscope. Our reference systems were the composites produced in a previous work [30], which made it possible to fully specify the impact of both the diatomaceous earth alone and the silanized diatomaceous earth on the characteristics of the obtained composites.

## 2. Materials and Methods

Polyamide 11 (Rilsan^®^ RE31840 Natural) was purchased from Arkema (Colombes, France). Diatomaceous earth (Perma-Guard, Bountiful, UT, USA) was derived from diatomite deposits. Silane APTES (3-Aminopropylotriethoxysilane) was purchased from Sigma-Aldrich (Saint Louis, MO, USA).

### 2.1. Preparation of Diatomite

The fractionation of amorphic diatomaceous earth was performed using the hydraulic method, according to the procedure presented in previous works [30,31], with the use of gravitational sedimentation. Fifteen kilograms of amorphic diatomaceous earth (particle size between 1 μm and 100 μm) was washed with demineralised water in order to separate particles of different sizes. We obtained three fractions of diatomaceous earth with disparate particle size distributions. In further testing, we used a fraction with a medium particle size (Fraction 2, particle size between 4 and 35 μm), where we eliminated the content of the smallest diatomite particles.

### 2.2. Preparation of Modified Diatomaceous Earth

We placed 1 kg of diatomaceous earth in a 14-L glass reactor equipped with a mechanical stirrer. Then, we added to the reactor 4 L of isopropanol, 10 mL of demineralised water, and 1% by weight of APTES (3-aminopropyl)trimethoxysilane, and we left it for 24 h, stirring continuously (300 rpm). Then, the mixture was poured into a separate container and left for sedimentation. Once the liquid was poured out from above the sediment, the diatomaceous earth was washed with demineralised water and put into a dryer at 60 °C for 24 h.

### 2.3. Extrusion of the Biocomposite

The biocomposite extrusion process was performed on a RHEO DRIVE 16 process line made by Haake PolyLab OS (Thermo Scientific) (Waltham, MA, USA). The process was carried out using a double-screw extruder. We added to the charge the mixture of PA 11 with basic diatomaceous earth, in portions (50 g), modified with the APTES at four concentrations of 2.5%, 5%, 10% and 20%, in that particular order. The materials became plasticised and mixed at the screws. The extruded substance was dried for 24 h at 70 °C and then milled in a plastics mill WANNER C17.26 sv (Łódź, Poland). We obtained 4 compositions of polyamide 11 with diatomaceous earth at concentrations of 2.5%, 5%, 10% and 20%. Other composites were extruded in a similar way. The extrusion parameters are shown in Table 1.

### 2.4. Injection Moulding–The Production of Standardised Measuring Paddles

The injection moulding procedure was performed on an e-victory 170/80 made by Engel (Schwertberg, Austria). Table 2 shows the process parameters. The mould temperature was maintained at 80 °C. We obtained standardised type 1A measuring profiles according to ISO 527 [32]. Finally, we produced 4 types of composites and a reference system (Table 3).

### 2.5. Characterisation Methods

For the mechanical analysis, we used dumbbell-shaped samples prepared according to EN ISO 527-1: 2020 [32] and EN ISO 178: 2010 [33] norms. The test was conducted on a universal testing machine INSTRON 5969 with a maximum loading force of 50 kN (Instron, Norwood, MA, USA). The transverse travel speed for tensile and flexural strength measurements was set at 5 mm/min.

The Charpy impact test with a notch was performed on an impact testing device, Instron Ceast 9050 (Instron, Norwood, MA, USA), according to ISO 179-1: 2010 [34]. The impact test was carried out according to PN-EN ISO 178-1 [33]. The notch was made by machining in the centre of the test sample according to ISO 179-1/1 eA [35]. The V-notch was angled at 45° and 2 mm deep. The side impact direction was applied in the line axis normal to the sample plane.

Dynamic mechanical thermal analysis (DMTA) was performed using a Q800 DMA (TA Instruments, USA) in dual cantilever mode according to ASTM D4065-01. From each composite, three rectangular specimens 60 mm in length and 10 mm in width were cut and used in the test. The analysis was conducted from 0 °C to 130 °C with a heating rate of 3 °C/min at a frequency of 1 Hz and an amplitude of 30 µm. The glass transition temperature (Tg) was determined from the peak value in the storage modulus using V4.5A TA Universal Analysis software.

Powder morphology was characterised by a scanning electron microscope (SEM, TM 1000 Hitachi, Tokyo, Japan) operating at an applied voltage of 5 kV. Before observations via SEM, the sample surfaces were sputtered with a gold-palladium layer for 90 s at an electric current of 10 mA and voltage of 2 kV.

The melt flow rate (MFR) was measured using the Instron CEAST MF20 melt flow tester, which accords with EN ISO 1133 [36], at 235 °C for a load of 2.16 kg.

Thermal properties of materials were studied by the DSC (differential scanning calorimetry) technique, using a Q1000 Differential Scanning Calorimeter (TA Instruments, New Castle, DE, USA). Samples with a weight of 8.0 ± 0.2 mg were placed in an aluminium hermetic pan. First, the samples were equilibrated at −90 °C, then heated to 230 °C with a scan rate of 10 °C/min, and cooled to −90 °C with a scan rate of 10 °C/min. Finally, they were heated again to 230 °C with a scan rate of 10 °C/min. The process was conducted in a nitrogen atmosphere. Using Universal V4.5A TA software, the glass transition temperature (Tg) was determined as the midpoint of the glass transition temperature range. The melting temperature was taken as the peak temperatures of cold crystallisation and melting, respectively. For the calculation of the composite crystallinity, the following formula was used, which is in accordance with the general methodology for DSC analysis of polymer composites [37].

$$X=\frac{H_{m}^{0}}{H_{0}}\cdot \frac{100}{100-a}$$

*a*—filler loading [wt%]

*H_m_*—measured melting enthalpy [J/g]

*H*_0_—melting enthalpy of fully crystalline PA11, 206 J/g [30].

The viscosity of the composites was determined with a capillary method, using a capillary rheometer Instron CEAST SR10 Smart RHEO 1000. The measurement was performed at 235 °C, and the capillary tube used was 1 mm in diameter and 20 mm in length. The viscosity was determined for 6 shear velocities: 10, 30, 100, 500 and 1000 [1/s].

The images of surfaces and fractures were made with a digital microscope KEYENCE VHX-7000 (KEYENCE INTERNATIONAL (BELGIUM) NV/SA) with a wide-angle zoom lens VH-Z100R with 100-fold magnification. The images were made using depth composition and 3D imaging features. We used full coaxial illumination.

Contact angle analyses were performed by the sessile drop technique (5 μL) at room temperature and atmospheric pressure with a Krüss DSA100 goniometer.

Surface gloss measurements were recorded at 23 °C according to the ISO 2813 [38] standard using a compact gloss meter (GM38, 3Color^®^, Narama, Poland) using 20°, 60° and 85° geometry. The device was calibrated between various specimen measurements using dedicated standard black glass. Three gloss unit (GU) values were recorded from each specimen and averaged. Before the measurements, the samples were stored in the same lighting conditions to prevent possible colour changes.

The phase identification and the relationship between the sediment fraction depth and its composition were determined using an X-ray diffraction (XRD) powder diffractometer Bruker AXS D8 Advance (Bruker, Karlsruhe, Germany) using CuKα lamp radiation and an Ni filter. X-ray diffractograms were recorded in the angular range of 5–80° (2Θ).

The measurements for hiding power were performed by placing samples of the prepared resin systems into the optical path between the light source (LED) and the UV-NIR spectrophotometer AvaSpec-Mini2048CL (Avantes, Louisville, CO, USA).

Fourier Transform-Infrared (FT-IR) spectra were recorded on a Nicolet iS50 Fourier transform spectrophotometer (Thermo Fisher Scientific, manufacturer Madison, WI, USA) equipped with an ATR unit (5000–80 cm^−1^).

For the washability test, two types of markers were used: a water-resistant permanent marker (green) and removable board markers in two colours (black, blue). Having applied a thin layer of each of the tested markers, the following test steps were planned:

- One-time wiping of the surface with a clean, dry cotton cloth,
- One-time wiping of the surface with a clean cotton cloth soaked with tap water,
- One-time wiping of the surface with a clean cotton cloth soaked with methanol,
- One-time wiping of the surface with a clean cotton cloth soaked with acetone.

The last step was not carried out as all traces of the markers had been removed previously by methanol.

The measurements for hiding power were performed by placing samples of the composites into the optical path between the light source (LED) and the UV-NIR spectrophotometer, an AvaSpec-Mini2048CL (Avantes, Louisville, CO, USA).

The size of the diatoms used to prepare the composites was measured with a Mastersizer 3000 (Malvern Instruments Ltd., Malvern, UK). The measurements were taken for samples in water suspension (Hydro EV attachment). The parameters of the measurements for the wet samples were a stirrer revolution speed of 2330 RPM and an ultrasound power of 70%. The tensile, bending and impact strength tests, rheological tests (MFR, viscosity), thermal tests (DSC, DMTA) and microscope observations were compared to the composites produced by direct injection moulding, which are described in a previous paper [30]. Additionally, the tests were complemented with a surface analysis, that is, the analysis of wetting angle and the test of gloss at the composite surface for all samples, whether or not they were modified by the APTES.

The particle size distribution of the filler was performed by Dynamic Light Scattering (DLS) on a Mastersizer 3000 instrument (Malvern Panalytical, Malvern, UK) with a Hydro EV measuring attachment.

## 3. Results and Discussion

### 3.1. Infrared Spectroscopy (FT-IR)

**Figure 3:**
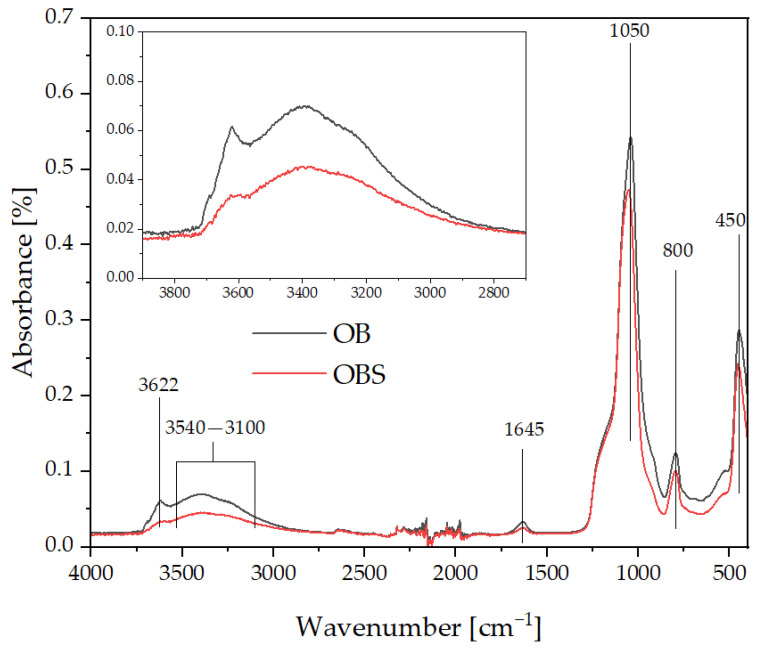
FT-IR spectra of neat, base diatomaceous earth (OB) and silanized diatomaceous earth (OBS).

Figure 3 shows the FTIR spectra of unmodified diatomaceous earth and fractionated diatomaceous earth silanized with APTES, used for modifying polyamide 11.

Both modified and unmodified diatomaceous earth are characterised by the presence of bands at 3622, 3540–3100, 1645, 1050, 800 and 450 cm^−1^. The silanization process did not produce new bands in the FT-IR spectrum, while a change in the intensity and peak maximum shift of the bands after the silanization process was observed.

The presence of a broad band in the range of 3540–3100 cm^−1^ is related to the presence of stretching vibrations of surface -OH groups, and a band at 1645 cm^−1^ being -OH bending vibrations of coordinated H_2_O molecules [39] or Si-OH silanol groups [40], while the band present at 3622 cm^−1^ is related to the O-H vibrations of adjacent SiOH groups forming intermolecular hydrogen bonds [41]. Silanization of diatomite resulted in a significant reduction of the O-H bands, both the main, broad one, and the sharp one at 3622 cm^−1^. The band present at 450 cm^−1^ is related to the muscovite present in the samples. The bands present at 1050 cm^−1^ and 800 cm^−1^ come from Si-O bond vibrations, as in the band in [42].

### 3.2. Particle Size Distribution

**Figure 4:**
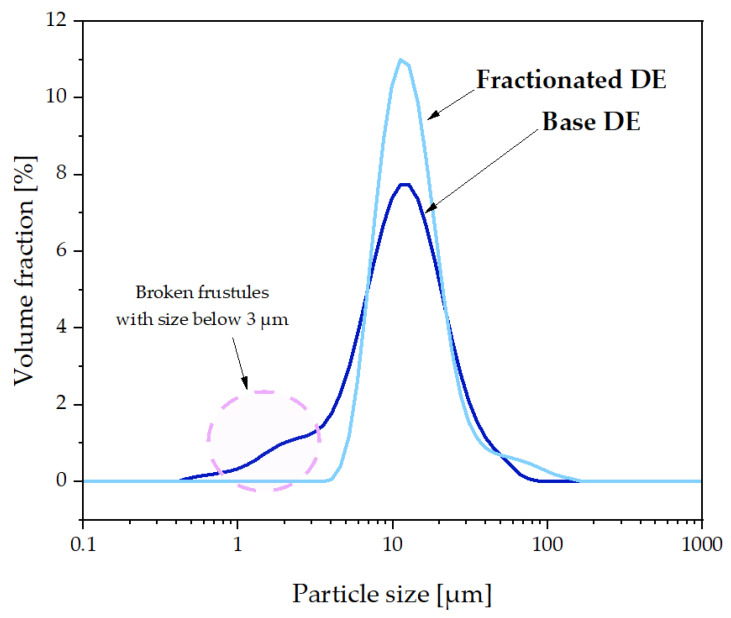
Particle size of the fillers, basic and fractionated diatomaceous earth.

Particle size distribution analysis was performed by Dynamic Light Scattering (DLS) for diatomaceous earth before and after the sedimentation fractionation process. The fractionation process allowed for the removal of the smallest diatomaceous earth particles, i.e., less than 4 μm, as shown in Figure 4. In addition, the effect of the sedimentation fractionation process on the tendency to form agglomerates is also observed, which can be seen as an increased proportion of particles with sizes in the 50–200 μm range. It is also observed that the largest amount of diatomaceous earth particles with a size of 7–12 μm is present, which is the main fraction of the material.

### 3.3. Crystallinity Analysis (XRD)

**Figure 5:**
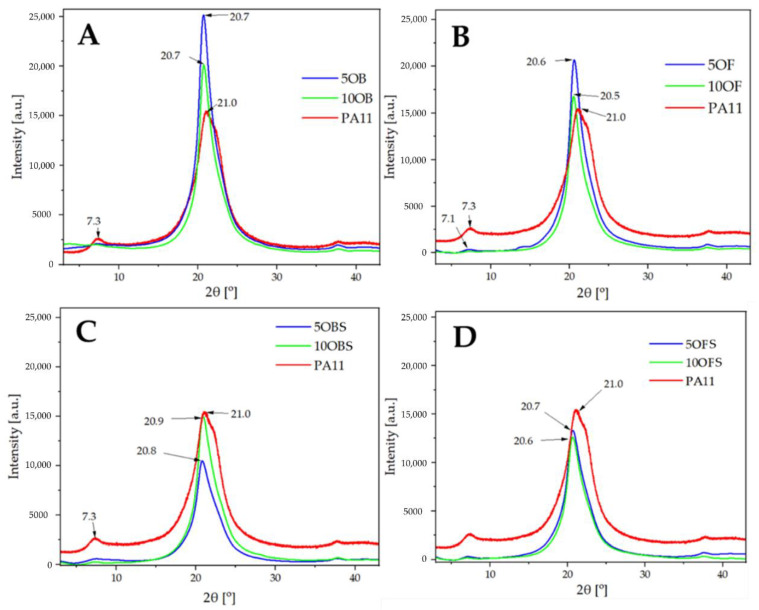
XRD PA11/diatoms with and without silane APTES; (**A**)—samples with base diatomite; (**B**)—samples with fractionated diatomite and APTES; (**C**)—samples with base diatomite; (**D**)—samples with fractionated diatomite and APTES.

XRD analysis of all studied materials was performed in order to investigate the effect of the diatomite filler on the crystallisation of the polymer matrix and to study the crystalline structure of neat PA11 processed under the same conditions. It is known that PA11 and other polyamide types are characterised by polymorphism, resulting in overlapping XRD reflexes, making the crystallographic analysis of composites based thereof a challenging task. Common polymorphs observed are α, γ, δ’ (smectic) and amorphous phases, and a high temperature δ phase forming above 95 °C (known as Brill transition temperature) in a reversible transition of α phase [43]. In addition, various crystalline phases have been reported in diatomite, including quartz, cristobalite, calcite, feldspar, kaolinite and vermiculite [44,45,46]. Analysis of neat PA11 (Figure 5) showed that the commonly observed α phase is present, as a reflex at 2θ of 7.3 is visible, corresponding to the (001) lattice, which is in agreement with available reports [43]. For a diffraction pattern of neat PA11 α phase, additional reflexes in 2θ of 20.0–20.5 and 22.9–23.5 ranges should be present, corresponding to the (200) and (010) + (210) lattices, respectively. In the studied case, a right-side broadened reflex at 2θ of 21.1 with another smaller maximum at 2θ ~22.3 is visible. This shift can be explained by the presence of an additional metastable γ phase, which is reported to give reflexes at 2θ of 21.6 and 22.4 [47]. The presence of some amounts of the δ’ smectic phase was also plausible, but the obtained data did not allow for its unambiguous confirmation.

In the case of non-silanized filler composites, both fractionated and non-fractionated diatomite, strong and well-pronounced reflexes of the δ’ smectic phase, were observed in each system, with the maximum at 2θ ~20.6. The presence of a coexisting α phase was confirmed by the still visible reflex at ~7.2; however, the abundance thereof decreased together with increasing filler content. It was initially thought that the application of the filler could have also caused changes in polymer crystallinity; however, DSC analysis (Section 3.6) revealed that the differences in the crystallinity index (CI) of the produced samples was barely noticeable. Therefore, the filler presence affects the selectivity of the PA11 α phase nucleation, while the obtained CI levels are comparable and, rather, the result of the injection moulding parameters kept constant for all the produced materials. The analysis of composites filled with APTES-silanized diatomite allowed us to draw the same conclusions, showing no particular effect of the filler surface treatment on its nucleation-inducing behaviour.

### 3.4. Test of the Composites’ Mechanical Characteristics

**Figure 6:**
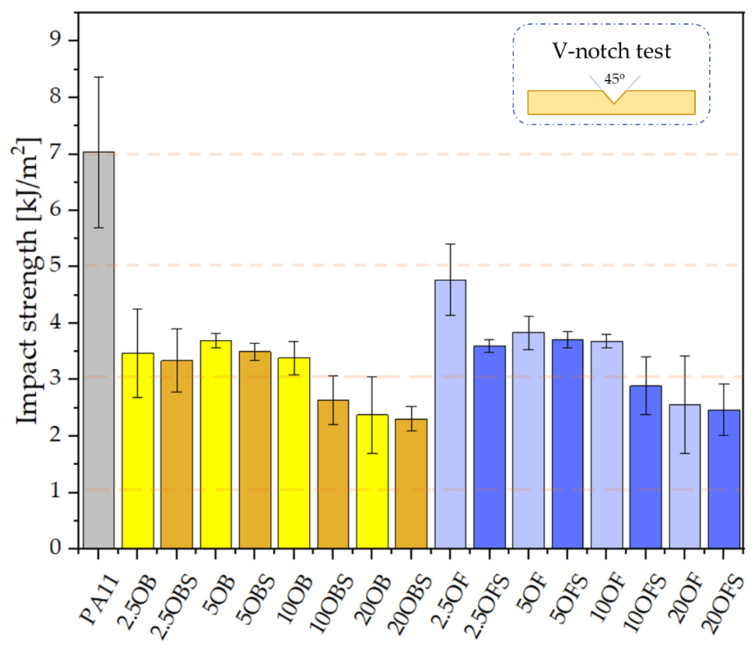
Impact strength of the composites after injection moulding: comparison of the composites containing diatomite filler with and without APTES silanization.

The notch impact test was conducted for the composites both with the addition of silane and, comparatively, without silane, which was presented previously in [30]. Neat polyamide was characterised by the highest impact strength amongst the tested samples, 7 kJ/m^2^ (Figure 6). The relatively low impact strength values arose from the high elasticity of the composites and from the requirement to test samples that were previously notched. For this reason, the impact strength values are lower than those recorded in the literature [48]. As in the previous work, we observed an impact of the size of diatomaceous earth particles on the composite impact strength. Composites containing fractionated diatomaceous earth showed a higher impact strength, but the differences were not too big. The silanization of PA11/DE composites in most cases caused a slight reduction in impact strength; a greater decline was recorded for the fractionated diatomaceous earth, while most of the modified composites showed a lower standard deviation, which means that the addition of silane had a positive impact on this aspect, i.e., probably on the filler dispersion in the polymer matrix, while for higher filling levels it could affect the secondary agglomeration of particles in the composite, which caused the slight reduction in impact strength.

**Figure 7:**
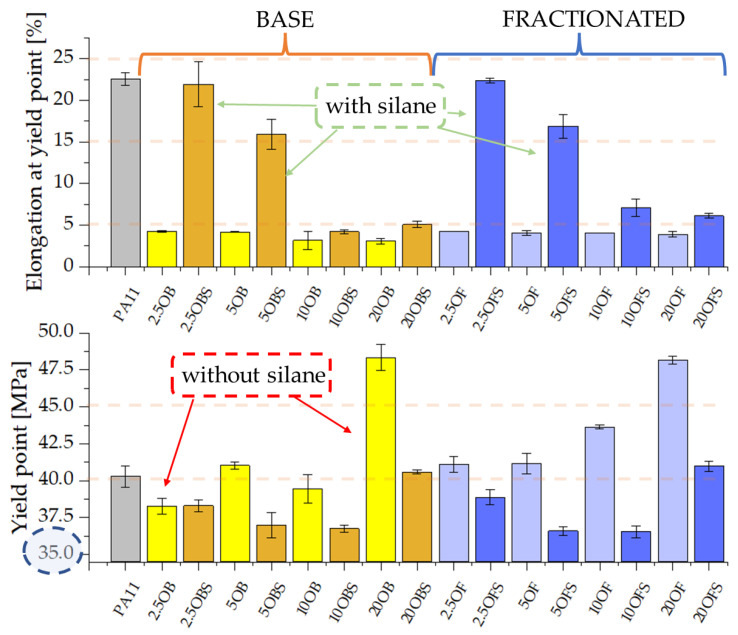
Elongation at yield point and yield point of PA11 composites contining silanized and non-silanized diatomite.

The silanization of diatomaceous earth had a very significant impact on the composites’ mechanical properties, in particular on the elongation at the yield point, which is shown in Figure 7. Composites containing pure diatomaceous earth show elongation at a yield point of around 5%, whereas, in particular for the low filling with silanized diatomaceous earth, we recorded an over 4-fold growth in that parameter irrespective of the pre-fractionation of the filler. The higher the diatomaceous earth concentration, the smaller the differences. The relationships presented above indicate that the addition of APTES increased the elasticity of the composites. That relationship might be associated with the fact that, if the level of filling the composite with diatomaceous earth is low (2.5%) and the content of the silane-based modifier is stable, the effect of silanization alone prevails over the effect of increased brittleness caused by the diatomaceous earth. As the filler concentration grows, the filler starts to prevail over the silanization effect, which consequently causes a decline of elongation at break. In contrast, the yield point was reduced after silanizing the filler, being lowest for systems with 10% concentration, whereas further filling of the composites caused the growth of the yield point.

**Figure 8:**
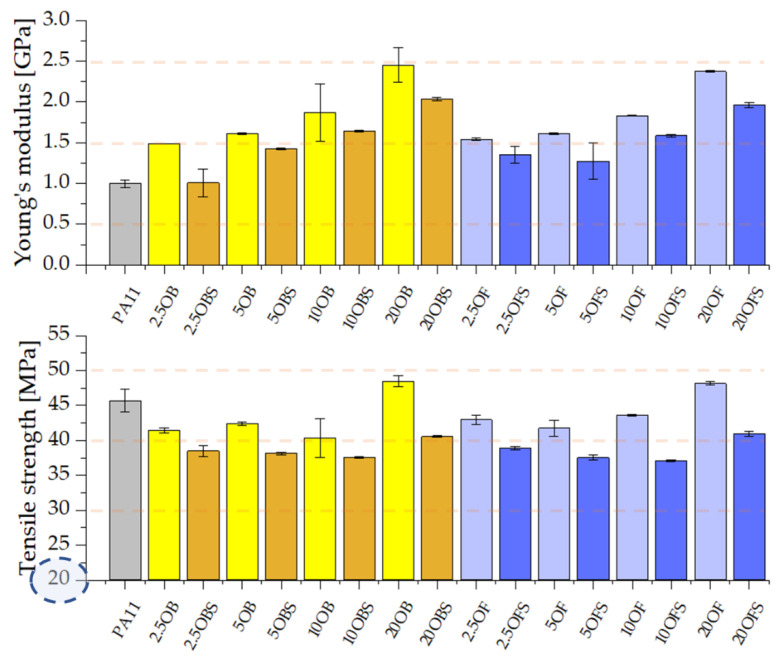
Young’s modulus and tensile strength of PA11 composites containing silanized and non-silanized diatomite.

**Figure 9:**
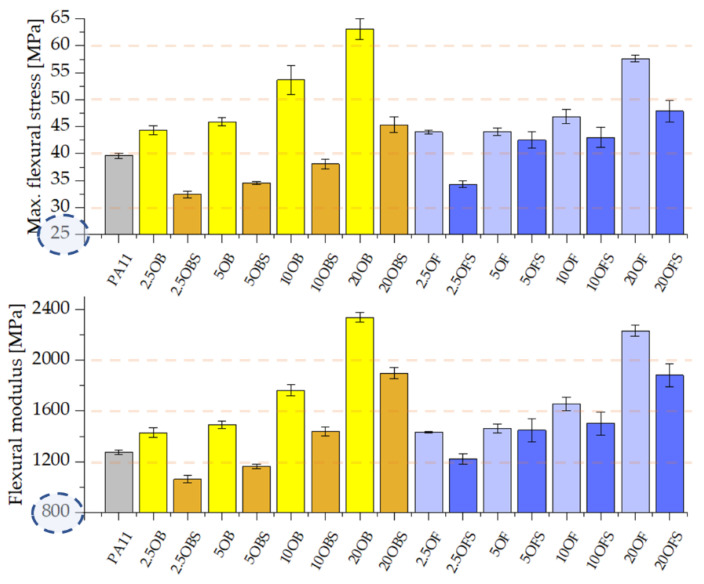
Maximum flexural stress and flexural modulus of PA11 composites containing silanized and non-silanized diatomite.

Composites that contain the silanized filler show somewhat lower values of Young’s modulus, tensile strength, flexural modulus or maximum flexural stress, but these parameters remain acceptable, particularly for high concentrations (Figure 8 and Figure 9). High-filled samples show values that are higher than those for the reference polyamide 11, and thus we can conclude that a high filling level (20%) is beneficial not only due to reduced costs of production of the composites but also due to the fact that they maintain acceptable mechanical parameters. Lower results, e.g., for the yield point of the composites, particularly those highly filled, may result from the fact that diatom agglomerates are formed in the composite, where the accumulation of APTES is higher, so there may also occur local cross bonds between the silane particles inoculated on diatomaceous earth [28]. Although it was tempting to correlate the observed differences between the mechanical properties and the crystalline polymorphism of neat PA11 and composite samples thereof, the literature data on the mechanical properties of the PA11 polymorphs was found insufficient to discuss the phenomenon of filler-induced crystallisation of PA11. The reports covered the XRD analysis of PA11-based composites; however, either no correlation between polymorphism and mechanical properties was discussed, or it was explained that the data did not allow us to discuss such a relationship [49,50].

### 3.5. Rheology—Melt Flow Rate (MFR) and Viscosity

**Figure 10:**
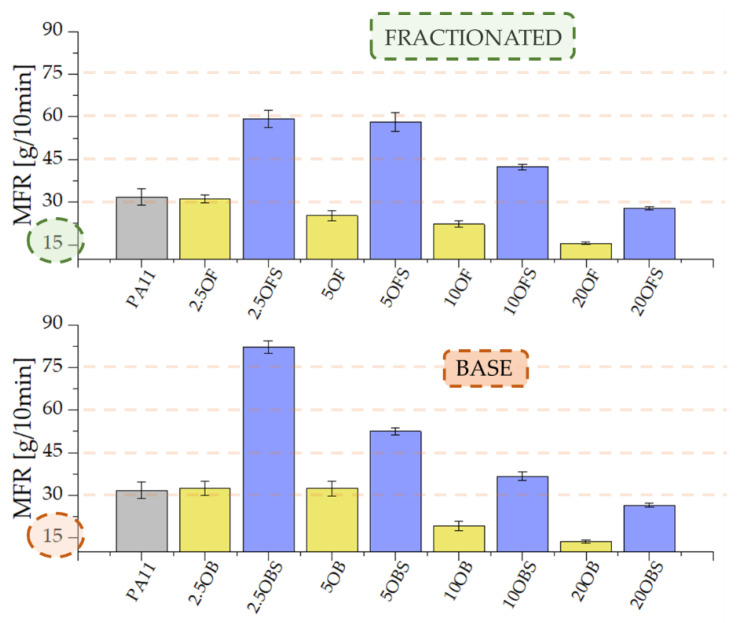
Melt flow rate (MFR) of the granulates of PA11/DE composites after injection moulding.

**Figure 11:**
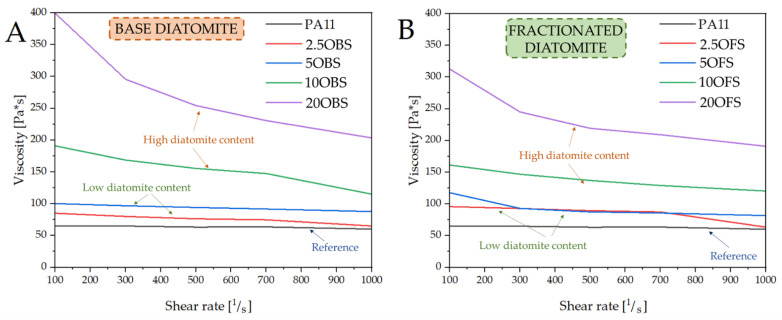
Viscosity of PA11/DE composites after injection moulding: (**A**)—PA11 and 2.5–20% OBS, (**B**)—PA11 and 2.5–20% OFS.

Silanization of diatomaceous earth positively affected the melt flow rate of PA11/DE composites. We observed considerable growth in processing parameters, especially at the lowest concentrations. As the filler concentration grows, the MFR reduction effect starts to prevail, which arises from the nature of the filler; however, we still recorded a twofold growth in MFR compared to the composites without silane (Figure 10). In earlier research [25], where the polymer matrix was formed by a polylactide, a similar effect was observed, where the MFR index grew even higher once silane was introduced to the system. The apparent dynamic viscosity of polyamide 11 was around 60–65 Pa·s regardless of the range of shear velocity (Figure 11), whereas the introduction of silanized diatomaceous earth to the systems caused a growth of that value along with increasing concentration, but at low DE concentrations the viscosity curve was similar to that of the reference sample. Upon exceeding 10% by weight of the filler, the viscosity at the initial values of shear velocity grew approximately twofold, particularly for the system containing basic diatomaceous earth. The functioning of diatomite and the filling of the composite with diatomaceous earth with a limited particle size distribution resulted in somewhat lower values of apparent dynamic viscosity. The added (3-Aminopropyl)trimethoxysilane acts as an agent that increases the melt flow rate (MFR), especially for low filling levels, and increases the viscosity of composites at high concentrations. The viscosity grew to around 50 Pa·s, depending on the shear velocity.

### 3.6. Thermal Analysis (DSC, DMTA)

**Figure 12:**
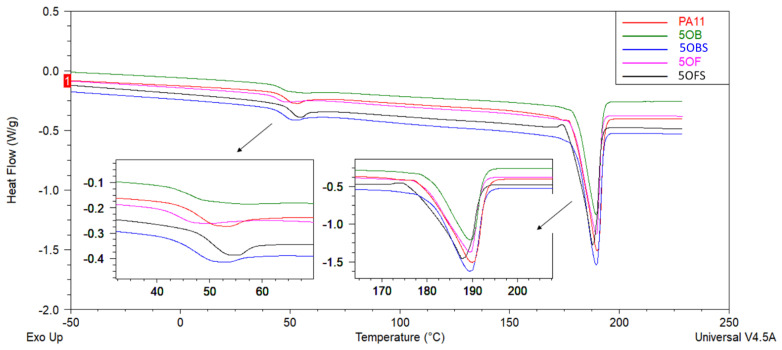
DSC curves of unmodified PA11 and composites, first heating; samples with 5% of diatoms.

**Figure 13:**
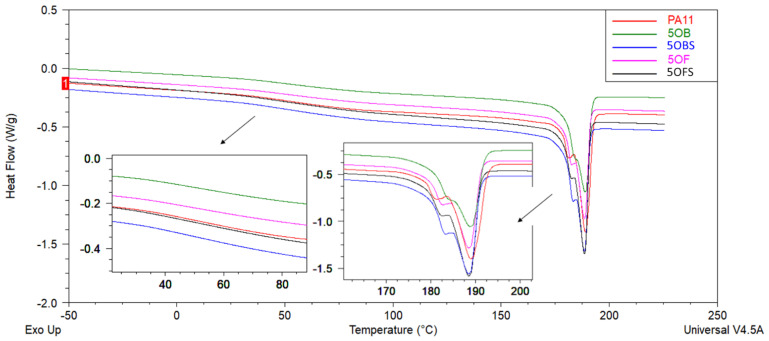
DSC curves of unmodified PA11 and composites, second heating; samples with 5% of diatoms.

**Figure 14:**
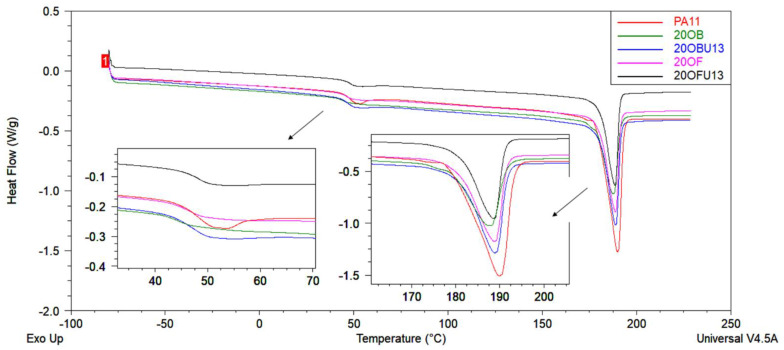
DSC curves of unmodified PA11 and composites, first heating; samples with 20% of diatoms.

**Figure 15:**
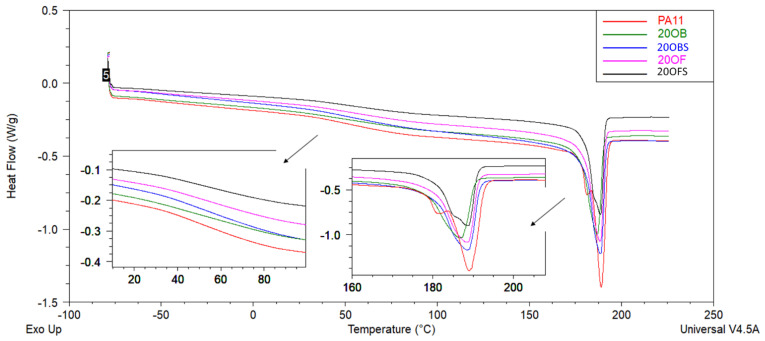
DSC curves of unmodified PA11 and composites, second heating; samples with 20% of diatoms.

DSC analysis was performed to investigate the thermal behaviour of the PA11 and PA11/diatomite composites. Figure 12, Figure 13, Figure 14 and Figure 15 show the DSC thermograms of PA11 and PA11/diatomite composites during the first and second heating cycles for 5% and 20% loading, respectively, and Table 4 and Table 5 present thermal characteristics of the materials with and without silane treatment obtained during two heating cycles. With increasing temperature, DSC curves show two thermal events: a glass transition (T_g_) near 50 °C, and a single endothermic melting event near 190 °C. It was found that the addition of diatomite did not affect T_g_ or T_m_ to any significant extent. On the other hand, in the previous work [30], a more significant drop in T_g_ was observed upon the addition of diatomite, showing that untreated diatomite presence causes formation of a filler–matrix interphase of weak interaction, with additional polymer freedom, while the filler treatment with APTES increases this interaction. The addition of diatomite caused a slight increase in the crystallisation temperature, showing some nucleation properties of the filler; however, silanization thereof caused no difference in this matter. Therefore, silanization does not affect the diatomite efficacy of PA11 nucleation, although it significantly affects the selectivity of nucleating the polymer towards either α or γ polymorph, as proven by XRD (see Section 3.3). For the second heating cycle, significant differences in PA11 behaviour were observed, mainly the reduction in T_g_ slope to the point where its exact determination was unfeasible. Additionally, both the unmodified PA11 and all 5% composites showed two melting peaks (T_m1_ and T_m2_) during the second heating cycle, and for 20% composites this double melting effect was still visible for all the materials by broadening and left-side shouldering of the endotherm signal, while being much better resolved for 20OFU13. While the main (larger) melting event was still observed at around 189 °C, the additional melting peaks were characterised by a maximum of around 182 °C, which was a result of formation of the abovementioned γ crystalline phase (smectic phase), accompanying the α phase melting at ~189 °C [47]. Depending on the processing conditions, PA11 may crystallise into different polymorphs that can be transformed from one to another. This double peak corresponded to the melt-crystallisation process of the γ phase to the α’ crystalline form of PA11. The secondary peak seemed to decrease when the additive content was increased in the composite material [51,52,53]. In addition, the glass transition temperature peak broadened considerably, and it was difficult to determine T_g_ from the DSC curves during the second heating. We did not observe a significant impact of the added (3-aminopropyl)tririmethoxysilane on thermal stability of the composites; however, as noted also in a previous work [30], the size of diatomite particles did have a slight importance and, for the fractionated diatoms, we observed an increase in temperature T_g_ by around 1 °C for the first heating cycle.

**Figure 16:**
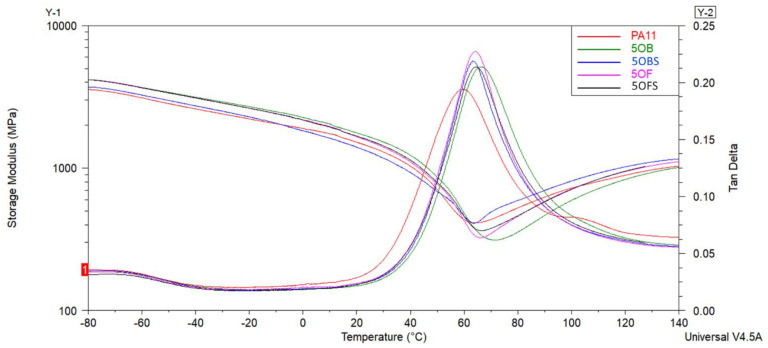
The storage modulus and tan δ curves of unmodified PA11 and composites; samples with 5% of diatoms.

**Figure 17:**
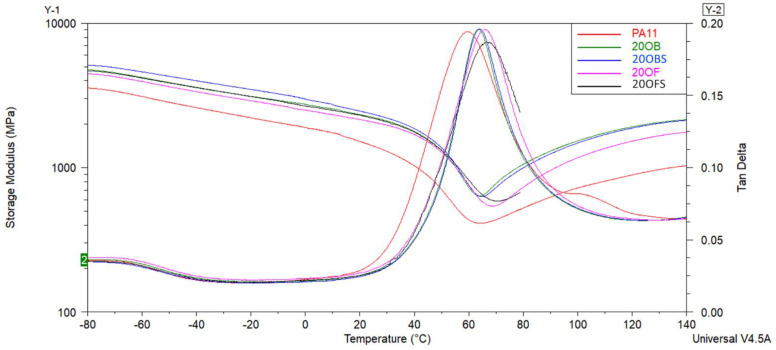
The storage modulus and tan δ curves of unmodified PA11 and composites; samples with 20% of diatoms.

The effect of the diatom concentration and addition of (3-aminopropyl)tririmethoxysilane silane on the viscoelastic behaviour of composites was studied by DMTA. The curves of the storage modulus (E′) and tan δ as the temperature’s function are shown in Figure 16 and Figure 17, while the obtained results are summarised in Table 6. The storage modulus or viscoelastic modulus corresponds to the elastic response of the material and is associated with the energy absorbed (stored) by the material and recovered in each load cycle. Generally, the increase in storage modulus is caused by mechanical viscoelastic stiffness raise [54]. The glass transition temperature (T_g_) was determined from the tan δ curve, which is defined as the ratio of the loss modulus E″ to the storage modulus E′. The peak of the tan δ curve occurs as a result of the relaxation of the polymer chains, and the glass transition temperature is attributed to it [55].

It can be seen that for neat PA11 the glass temperature was under 60 °C. In the presence of 20 wt% diatoms and (3-aminopropyl)tririmethoxysilane, T_g_ increased to 66.9 °C. It was found that the addition of diatoms with different sized frustules and silane shifted the Tg peak towards higher values of about 5 °C. This confirms a good dispersion of diatoms in the PA11 matrix, which limits the polymer chains’ mobility and causes mechanical viscoelastic stiffness to increase. A similar tendency was confirmed by other researchers [54]. The addition of APTES silane did not change the glass temperature much, which was related to not affecting the polymer chains’ movement. These results were correlated with the obtained mechanical parameters. The addition of silane did not increase Young’s modulus and tensile strength composites relative to chemical unmodified materials.

Looking into the presented curves, it should also be noted that the addition of diatoms into the PA11 polymer allowed for obtaining stiffer material with higher values of storage moduli investigated at room temperature and tan δ value due to the stiffening effect of the reinforcement. As observed, the increase of the diatom content tends to increase the value of E′ from 1409 MPa to 2340 MPa, suggesting an improvement in the material’s mechanical viscoelastic performance. At the same time, tan δ presented a slight increase for composites with 20 wt% of the diatom concentration, probably due to reducing the polymer’s relaxation caused by the reinforcing effect of diatoms that hinders the mobility of the PA11 molecular chains [54]. In comparison, Stoclet et al. indicated that the increase in the clay content and improvement of its dispersion involves an enhancement of thermomechanical properties of polyamide 11 nanocomposites [51].

### 3.7. Surface Properties

**Figure 18:**
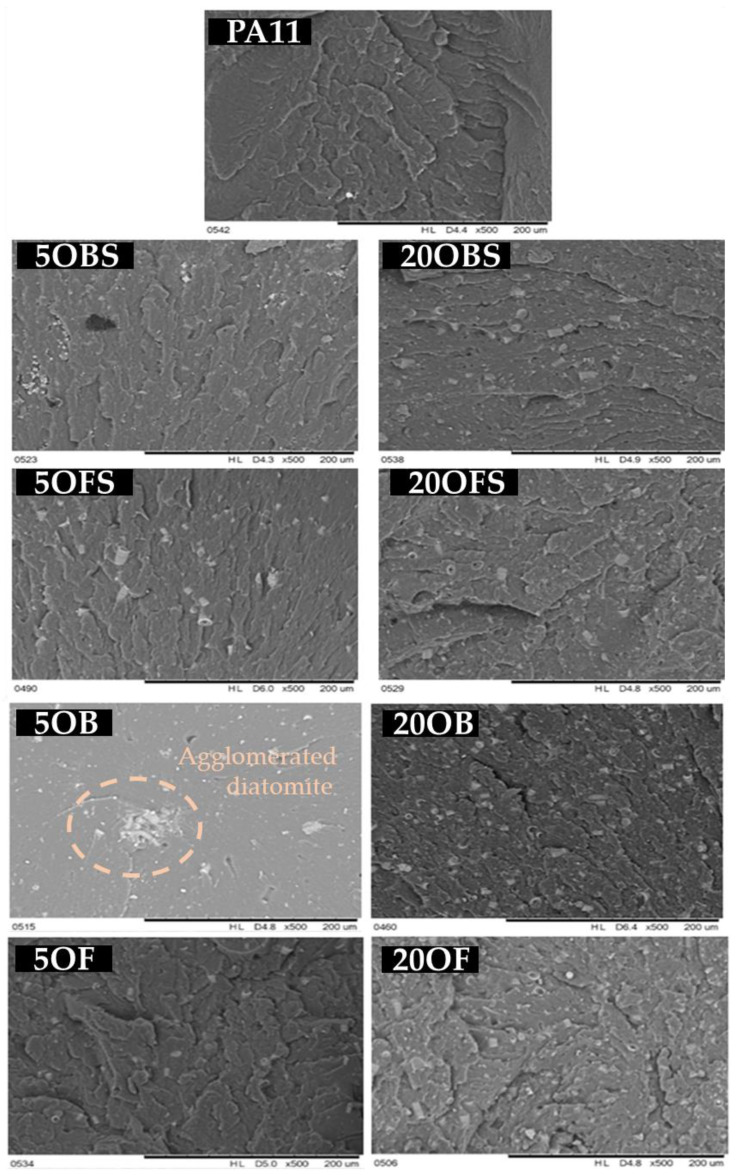
SEM images of composite fractures after testing the notch impact test; comparison to the systems without silane APTES.

Composite fracture imaging showed a relationship between both the method of preparing the filler and the tendency for secondary agglomeration of particles and between its concentration and the distribution of agglomerates in the polymer matrix (Figure 18). The higher the filler concentration, the more clusters of diatom particles were present, which was observed for both the basic and the fractionated diatomaceous earth. Basic diatomaceous earth showed a greater tendency towards secondary agglomeration than fractionated diatomaceous earth, which was due to the presence of particles of various sizes. Based on the comparison of SEM images showing the fractures of composites without silane, we found that it had an impact on the structure of the surface. The silanization of diatomaceous earth made the composite surface smoother, regardless of the fraction used and of its concentration. In addition, the share of grooves visible at the fracture declined.

**Figure 19:**
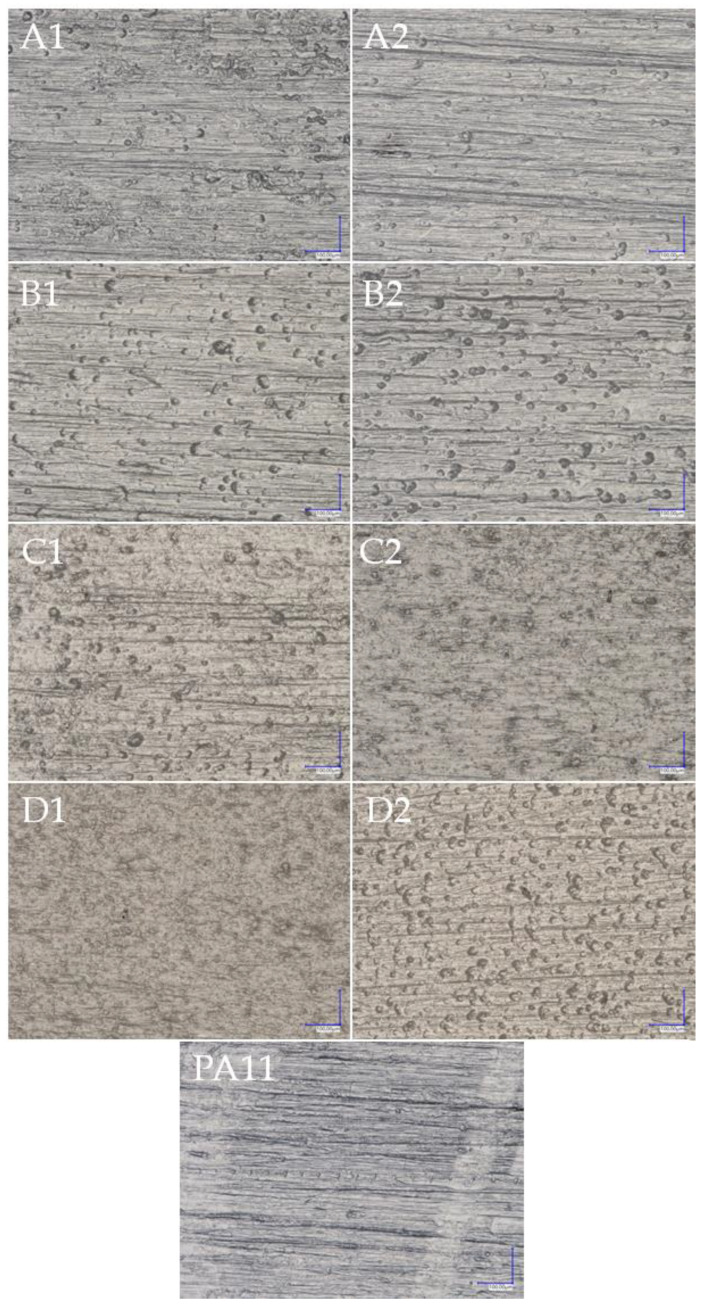
Surface of the composites; (**A1**)—2.5OBS, (**A2**)—2.5OFS, (**B1**)—5OBS, (**B2**)—5OFS, (**C1**)—10OBS, (**C2**)—10OFS, (**D1**)—20OBS, (**D2**)—20OFS, (**PA11**)—Polyamide 11.

**Figure 20:**
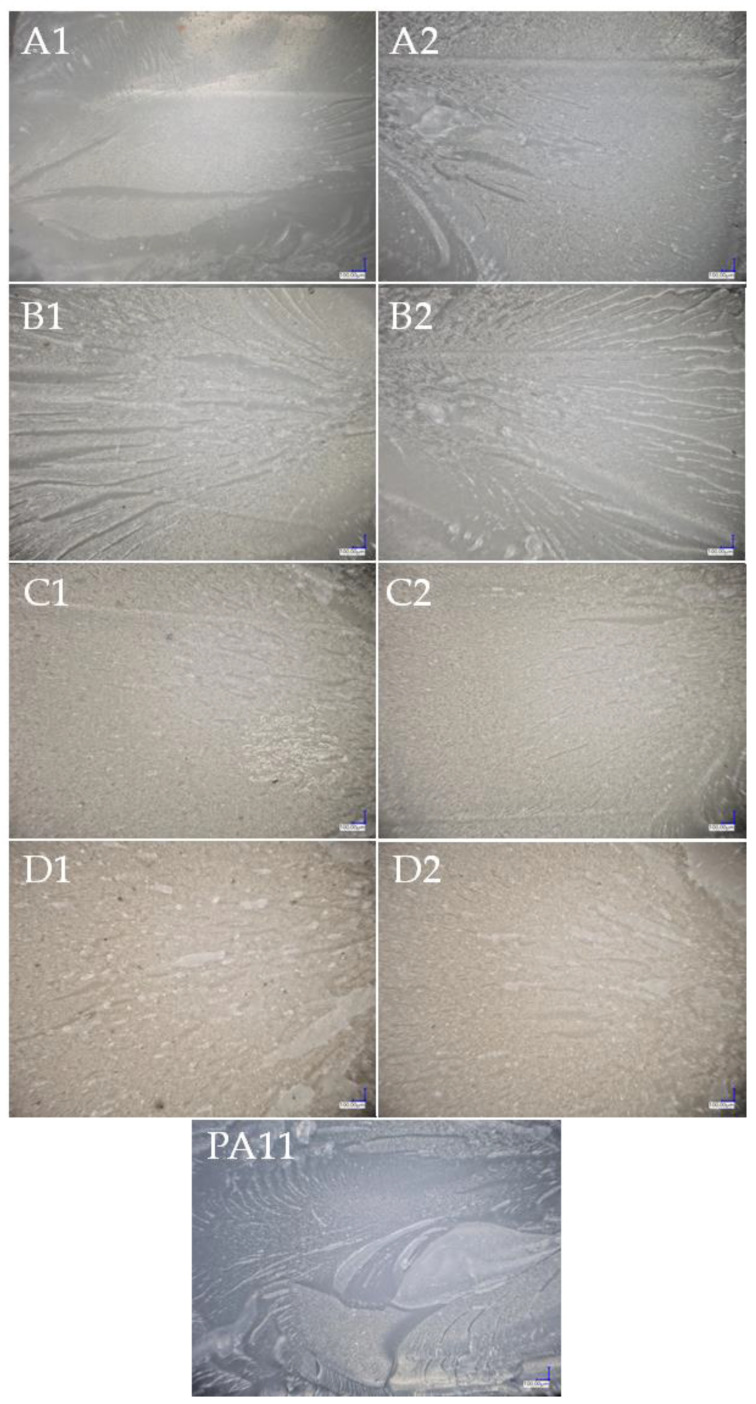
Fractures of the composites after the notch impact test; (**A1**)—2.5OBS, (**A2**)—2.5OFS, (**B1**)—5OBS, (**B2**)—5OFS, (**C1**)—10OBS, (**C2**)—10OFS, (**D1**)—20OBS, (**D2**)—20OFS, (**PA11**)—Polyamide 11.

**Figure 21:**
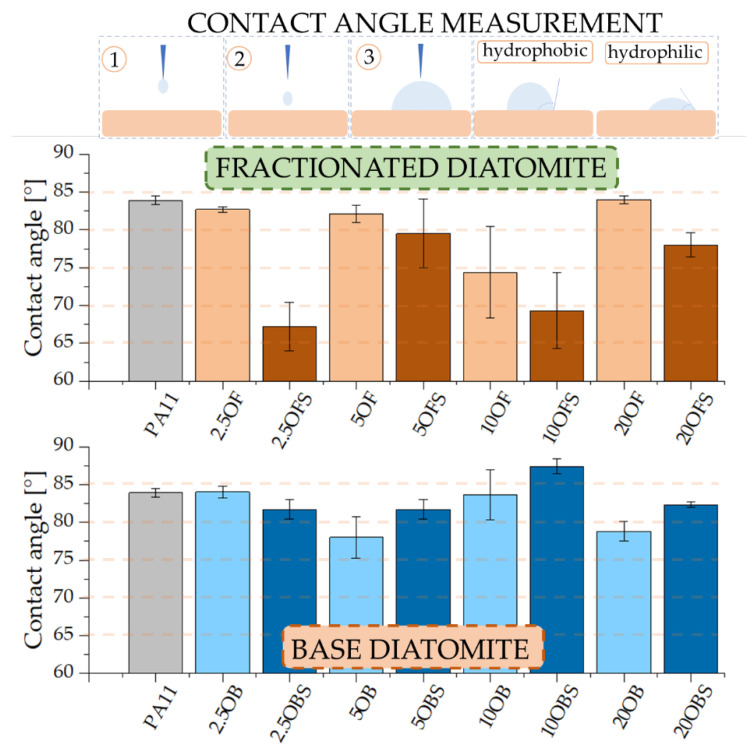
Wetting angle.

Images of the surfaces and fractures of the composites with an optical microscope were made (Figure 19 and Figure 20). Along with the growing filler concentration on the surface of the composites, we observed an increasing non-uniformity in colour due to the presence of hydrophilic centres (black spots) on the composite surface, which was in turn directly attributable to the values of the wetting angle and the hydrophobic-hydrophilic properties of the composites (Figure 21 wetting angle). In addition, there were differences in colour between the composites filled with basic and fractionated diatomaceous earth. This was caused by eliminating the systems of the smallest DE particles, which gave the composite an even, uniform colour, whereas larger, agglomerated particles were visible, as the dark spots discussed above. That effect was also slightly visible in the images of the composite fractures, while much larger differences were noticed on the surface of the samples. For the fractures, it was easier to see the colour difference arising from the mere concentration of the filler, so as the concentration grew, the colour of the composite changed towards that of diatomaceous earth alone, with a gentle difference due to the filler particle size.

The gloss tests were carried out both for the composites modified with silanized diatomaceous earth and for the composites without U-13 to make a comparison, as presented in Table 7. The test was carried out at 60° geometry. Neat polyamide 11 is characterised by a surface gloss of 31.5 GU, which makes it a medium gloss system. The addition of diatomaceous earth reduced the gloss, which decreased linearly along with the growing concentration of the filler having the form of basic diatomaceous earth. The modification of base systems with silane further reduced the gloss and, for the high-filled samples, the formation of systems with a near-matte surface. An exception is the system filled in 5%, subjected to silanization, as in this case we can observe a slight growth in gloss value, which is also visible for the fractionated sample 5OF. In this case, at the initial abrupt increase of gloss and achieving the limit for system 5OF, the values start declining along with further growth of the filler. It is similar after modification with APTES, but in this case the limit filling value turned out to be 10%, and each addition of silane caused a gloss reduction and the formation of systems with surfaces approaching matte.

The addition of APTES enabled us to obtain a hydrophilic composite system. The tests of hydrophobic–hydrophilic properties (Figure 21) showed that neat polyamide 11 had a wetting angle of around 84°, making it hydrophilic. This was similar to composites filled with diatomaceous earth, whose surfaces were also hydrophilic. Those based on polylactide were hydrophilic systems. The addition of base, silanized diatomaceous earth in most cases (except 2.5% loading), caused a slight growth in surface hydrophobicity, which might have been due to the presence of smaller filler particles, which do not exist in fractionated DE, and to the combination with APTES, which is likely to interact more effectively with smaller particles. The silanization of fractionated diatomaceous earth and the elimination of the smallest particles allowed us to obtain a more hydrophilic composite system compared to the others, but we found no relationship between the filler concentration and the wetting angle values.

**Figure 22:**
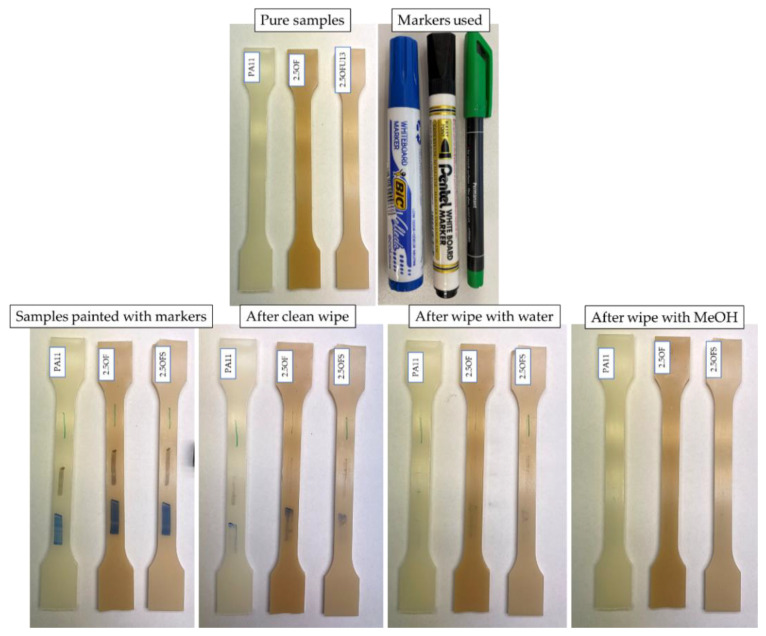
Washability test.

Washability tests were conducted (Figure 22) to determine whether PA11/diatoms are useful in industrial applications, for example in the overprinting of foils and packaging materials. We selected a composite characterised by the lowest degree of surface hydrophobicity (2.5OFS) and, for reference, a corresponding composite without silanization (2.5OF) and pure Polyamide 11 (PA11). The test showed that, after the first step of the washability test conducted on the surface of composite 2.5OF (without silanization), the green (permanent) marker was partially removed, which did not occur for the other samples; additionally, for that composite, we also found that the removable board marker (blue) remained on the surface to the greatest extent. Despite the much lower wetting angle for the silanized sample compared to pure polyamide 11, after using water, the trace of the permanent marker remained unchanged and had a similar intensity. Only methanol used to clean the surface completely removed each type of marker used. Based on the described tests, we concluded that a silanized composite showed higher resistance to surface washing than a composite containing pure diatomaceous earth, despite the difference in wetting angle, which was 16° on average, whereas pure polyamide 11 and composite 2.5OF, despite the nearly identical hydrophobicity level (~84° and 83°, respectively), showed significant differences in the behaviour of their surfaces when coloured.

**Figure 23:**
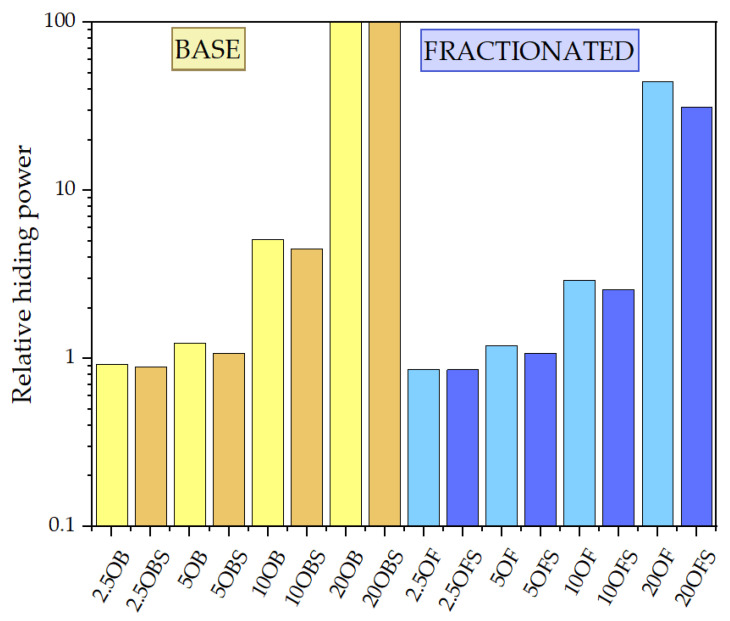
Relative hiding power.

**Figure 24:**
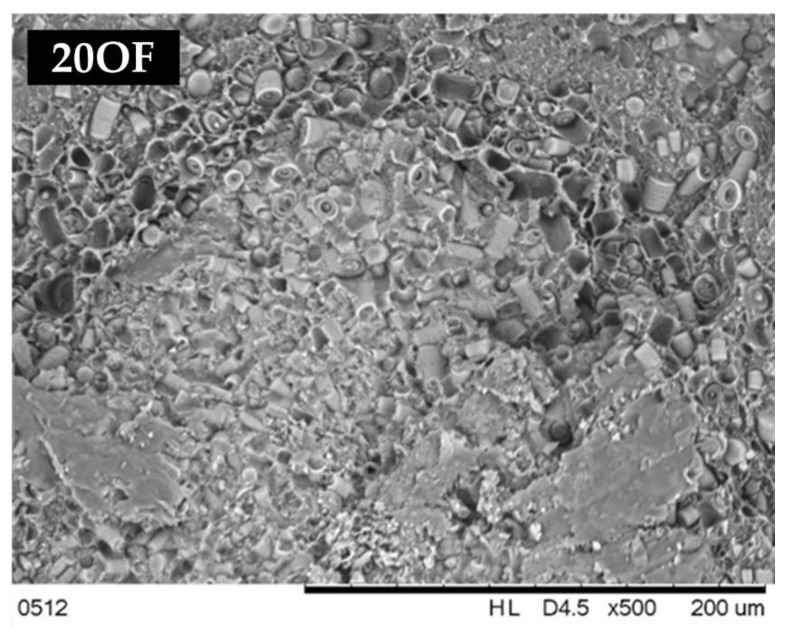
An SEM image of a composite containing 20% of basic diatomaceous earth.

**Figure 25:**
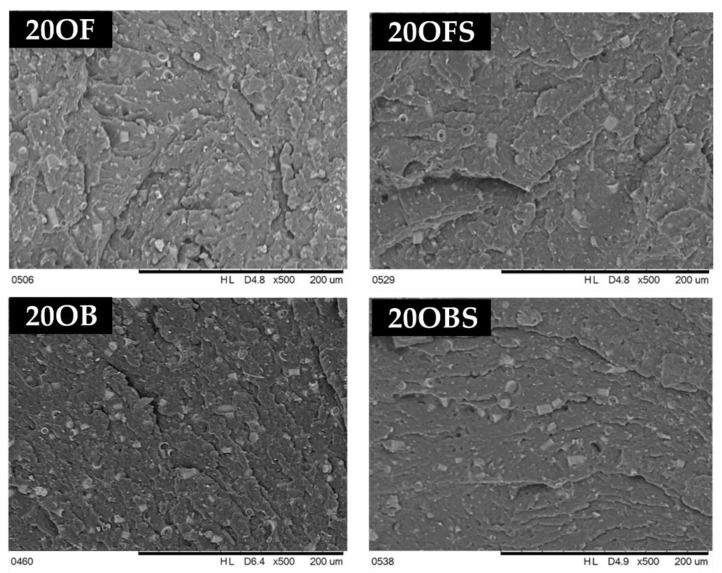
SEM images of composites containing 20% silanized and non-silanized diatomaceous earth.

The relative hiding power tests (Figure 23) of the composites indirectly permitted us to determine the composites’ colouring degree and transmittance. The relative hiding power increased along with the growing filler concentration, but that increase was not so significant up to 10% DE. In the case of composites modified with basic DE, the maximum recorded relative hiding power was around 5%, and 3% for fractionated DE, which indicates that the elimination of the smallest particles (<3 μm) increased the transmittance of the composites (Figure 4), implying a change in colour in this case. Composites filled with base diatomaceous earth at 20% showed the lowest relative hiding power values, while for fractionated DE, they were lower by half, which means that the effect of absence of the smallest particles was visible considerably better at higher concentrations. Figure 24 shows an SEM image of a composite containing 20% basic diatomaceous earth without silanization, which was made in a random location at the fracture of the sample. We observed large amounts of agglomerated diatomaceous earth containing broken and unbroken frustules. The filler agglomeration in the polymer matrix had a direct impact on the very high values of relative hiding power due to the hindered permeation of rays through the sample. Each modification of the systems with APTES reduced the relative hiding power, which was best visible for systems 20OF. The SEM images presented in Figure 25 show composites containing 20% of the filler. We observed a considerably higher dispersion of the filler in the polymer matrix, implying a lower hiding power for composite surfaces, which applies in particular to the systems with fractionated diatomaceous earth.

## 4. Conclusions

The tests allowed us to make conclusions on the effect of diatomaceous earth’s introduction into PA11 on the number of properties of such fabricated composites, as well as the impact of diatomite hydraulic fractionation and silanization. The addition of the diatomite filler results changed the crystalline structure of PA11 and the formation of δ’ smectic phase, as opposed to α + γ phases for neat PA11. The modification of diatomaceous earth favourably affected the elongation at the break of PA11/DE composites; we observed a significant improvement in their elasticity, particularly at low filler concentrations. The addition of APTES also contributed to a considerable improvement in the composites’ processing capacity, particularly at low filler concentrations, whereas, along with the growing content of DE, the effect brought by the characteristics of diatomaceous earth started to prevail. With the silanization of fractionated diatomaceous earth, we obtained a hydrophilic composite system, while the addition of APTES to basic diatomaceous earth increased the hydrophobic–hydrophilic properties of the surface. Depending on the desired parameters of the composite, the system properties were controlled by silanizing fillers of various particle sizes. On the surface of the composites, many hydrophilic centres were visible, the amount of which grew along with the increasing filler concentration. The centres had a direct impact on both the morphology of the surface of the composite and its hydrophobic–hydrophilic properties.

## Figures and Tables

**Table 1:** Extrusion parameters.

**Temperature at Heating Zones (°C)**	**Nozzle**	**TS6**	**TS5**	**TS4**	**TS3**	**TS2**	**TS1**
	235	250	250	255	255	250	250
**Torque, M (Nm)**		**Nozzle Pressure, P50 (bar)**			**Revolutions per Minute, N (1/min)**		
20–78		0.6			14		

**Table 2:** Injection moulding parameters.

**Temperature [°C]**	**Nozzle**	**Zone 3**	**Zone 2**	**Zone 1**	**Traverse**
	215	220	215	210	40
**Mold Closing Force (kN)**	**Clamping Time (s)**		**Cooling Down Time (s)**	**Screw Diameter (mm)**	
800	4		30	25	

**Table 3:** Composites obtained for testing.

Biocomposite Type	Filler Concentration	Abbreviation
PA11 + original diatoms + 1% wt. APTES[a]	2.5%	2.5OBS
	5%	5OBS
	10%	10OBS
	20%	20OBS
PA11 + fractionated diatoms + 1% wt. APTES[a]	2.5%	2.5OFS
	5%	5OFS
	10%	10OFS
	20%	20OFS
PA11	-	PA11

a—APTES was applied in 1 wt% ratio of the diatomite.

**Table 4:** Thermal characteristics of PA11 and composites with silane.

	1st Heating				Cooling		2nd Heating			
Sample	T_g_ (°C)	T_m_ (°C)	H_m_ (J/g)	Χ (%)	T_c_ (°C)		T_m1_ (°C)	T_m2_ (°C)	H_m_ (J/g)	Χ (%)
PA11	48.7	190.0	56.9	27.6	163.9		181.4	189.0	51.6	25.0
5OBS	47.9	189.4	52.0	26.6	167.8		183.4	188.5	50.5	25.8
5OFS	49.8	188.7	48.8	24.9	167.8		182.7	188.5	51.7	26.4
20OBS20OBU13	46.9	188.9	44.3	26.9	169.5		-	188.5	45.7	27.7
20OFS	48.2	188.5	34.6	21.0	169.2		-	188.6	32.1	19.5

**Table 5:** Thermal characteristics of composites without silane obtained in a previous work [32].

	1st Heating				Cooling		2nd Heating			
Sample	T_g_ (°C)	T_m_ (°C)	H_m_ (J/g)	Χ (%)	T_c_ (°C)		T_m1_ (°C)	T_m2_ (°C)	H_m_ (J/g)	Χ (%)
PA11	48.7	190.0	56.9	27.6	163.9		181.4	189.0	51.6	25.0
5OB	46.8	189.5	44.6	22.8	167.6		182.6	189.0	45.0	23.0
5OF	44.0	189.5	48.7	24.9	167.1		182.8	188.6	50.3	25.7
20OB	44.6	187.6	36.4	22.1	167.8		-	186.6	44.1	26.8
20OF	46.6	188.7	39.2	23.8	169.7		-	188.4	44.7	27.1

**Table 6:** Summary of the determined values of the glass transition temperature from tan δ curves and storage modulus E′ curves.

Sample	T_g_ (°C)	E′ (MPa)	tan δ
PA11	59.6	1409	0.194
5OB	66.4	1649	0.214
5OBS	63.2	1291	0.219
5OF	64.2	1534	0.227
5OFS	64.2	1560	0.214
20OB	63.4	2222	0.196
20OBS	64.0	2340	0.196
20OF	65.8	2062	0.196
20OFS	66.9	2190	0.187

**Table 7:** Gloss on the composite surface measured at 60° geometry.

	Gloss 60° [GU]	SD [GU]		Gloss 60° [GU]	SD [GU]		Gloss 60° [GU]	SD [GU]		Gloss 60° [GU]	SD [GU]
2.5OB	24.53	0.50	2.5OBS	22.20	0.36	2.5OF	23.00	0.08	2.5OFS	22.97	1.13
5OB	21.90	0.54	5OBS	22.30	1.12	5OF	35.70	1.13	5OFS	20.70	0.36
10OB	17.50	2.12	10OBS	21.30	1.45	10OF	26.10	0.75	10OFS	27.03	0.62
20OB	15.07	0.74	20OBS	12.47	0.17	20OF	16.57	0.12	20OFS	15.47	0.42
PA11				31.50				1.93			

## Data Availability

Not applicable.
